# Advancing Diabetic Foot Ulcer Care: Focus on the Post‐Healing Transition Phase

**DOI:** 10.1111/1753-0407.70173

**Published:** 2025-11-28

**Authors:** Kamran Shakir, Hadi Sarlak, Sicco A. Bus, Giulia Rogati, Alberto Leardini, Lisa Berti, Paolo Caravaggi

**Affiliations:** ^1^ Department of Biomedical and Neuromotor Sciences Alma Mater Studiorum ‐ Università di Bologna Bologna Italy; ^2^ Movement Analysis Laboratory and Functional Evaluation of Prostheses IRCCS Istituto Ortopedico Rizzoli Bologna Italy; ^3^ Department of Rehabilitation Medicine Amsterdam UMC, University of Amsterdam Amsterdam the Netherlands; ^4^ Program Rehabilitation Amsterdam Movement Sciences Amsterdam the Netherlands; ^5^ Physical Medicine and Rehabilitation Unit IRCCS Istituto Ortopedico Rizzoli Bologna Italy

**Keywords:** diabetic foot ulcers, footwear, offloading, plantar tissue, recurrence, remission

## Abstract

The recurrence of diabetic foot ulcers (DFUs) brings significant morbidity to people with diabetes and adds to healthcare costs. According to current guidelines, a DFU is considered healed when it is re‐epithelialized, and the wound closure is maintained for around 2 weeks. However, recent literature suggests that the mechanical properties of the underlying plantar tissues remain altered, thus making the foot vulnerable to recurrence. The period lasting several weeks post‐DFU healing can be termed the ‘transition phase’. This review aimed at exploring this critical phase, with an emphasis on the roles of tissue mechanics and current offloading strategies. An extensive search was performed on PubMed to identify studies on the mechanical properties of the tissues and preventative strategies in the post‐healing period. Following the analysis of titles and abstracts, 57 studies met the inclusion criteria and were included in the review. The analysis of the literature revealed that studies are primarily focused on offloading interventions during the remission phase to mitigate long‐term ulceration risks. The physiological needs of the still‐vulnerable plantar soft tissue after DFU healing are seldom assessed, and no strict differentiation between re‐ulceration and recurrent ulceration has been found. According to this review, there is a need for developing beyond state‐of‐the‐art solutions targeting pressure relief and footwear adherence, which consider the varying physiological conditions of the skin and underlying tissue in the transition phase, and the different risk categories and independent risk factors.

## Highlights

A new approach is needed for developing advanced offloading solutions that go beyond the state‐of‐the‐art, particularly focusing on the ‘transition phase’. This phase begins immediately after the ulcer heals, lasts for several weeks, and is characterized by high‐pressure offloading requirements and changing tissue properties.

## Graphical abstract

A model of diabetic foot ulcer management: from ulcer development to remission. Literature indicates that further research is needed to understand the transition phase for developing advanced offloading modalities.

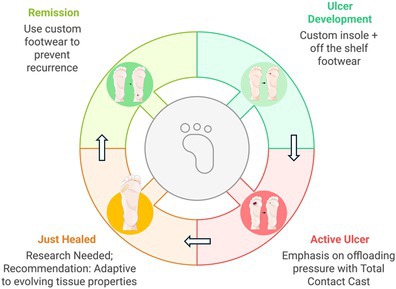

**Abbreviations**
DFU: diabetic foot ulcer
DM: diabetes mellitus
LOPS: loss of protective sensation
MTH: metatarsal head
PAD: peripheral artery disease

## 1 Background

Diabetes mellitus (DM) is a pandemic characterized by persistently high blood glucose levels, which may lead to physiological and biomechanical changes in the body [1]. In the foot, DM causes the thickening and stiffening of collagen‐rich plantar soft tissues, potentially leading to abnormally high plantar pressure during walking and daily activities [2]. Other factors, such as limited joint mobility, commonly found in people with DM, have also been shown to increase regional plantar pressure [3]. The elevated plantar pressure, coupled with diabetic complications like peripheral neuropathy, loss of sensitivity, and foot deformity, may lead to diabetic foot ulcers (DFUs) [4, 5].

DFUs are refractory and require a multi‐modal approach for healing, including callus debridement, vascular assessment and intervention if ischemic, infection control if infected, wound dressing to maintain a moist wound environment, monitoring and managing the blood glucose levels, and arguably the most relevant, pressure offloading of the affected plantar regions [6]. A recent meta‐analysis showed that it requires, on average, 50 ± 31 days for DFUs to heal [7]. After healing, DFUs are prone to recurrence [8]. The history of a plantar DFU is a strong risk factor for recurrence [9]. Studies suggest that nearly two out of five people with DM will experience a DFU recurrence within 1 year from healing of the first DFU and almost three out of five within 3 years [10, 11, 12, 13, 14]. In most cases, the recurring DFU is at an anatomical location different from the previous DFU [15, 16]. Studies have shown that, in the heel and medial forefoot, a second DFU is twice as likely to occur at a new position as compared to the exact anatomical position of the initial DFU [15]. This elevated recurrence rate highlights the need to understand the fragile condition of the foot after initial healing.

Following the closure of the epidermal layer of the lesion, the skin and the underlying tissue are still vulnerable and prone to damage if not properly managed; thus the likelihood of recurrence of DFU is high. The mechanical properties of the plantar skin and soft tissues in diabetic feet are different from those of non‐diabetic feet [17, 18, 19]. This difference is further exacerbated post‐ulcer [19] as the plantar skin becomes thinner and the lack of proper blood supply and underlying neuropathy causing loss of protective sensation, significantly impairs the skin's ability to withstand high pressure. Thus, while the primary DFU is treatable, sustained protection from subsequent DFUs is not guaranteed. Therefore, it's advisable to consider the person to be in remission of DFU [13] rather than healed. This change in terminology signals to the people with DM that they will require ongoing follow‐up and preventive care throughout their lives to avoid severe complications in the future. This concept of DFU remission calls for structured prevention strategies tailored to patient‐specific risk levels.

According to the International Working Group on the Diabetic Foot (IWGDF) guidelines, people with DM can be classified into four risk categories, and specific prevention and offloading protocols are recommended for each risk category [20, 21]. Despite advancements in these targeted healing protocols for secondary prevention, the global rate of DFU recurrence has not significantly decreased [22]. Studies have shown that recurrences can occur as early as the first few months after DFU healing [13, 23]. This data indicates that the current practice, including preventive strategies such as current foot offloading methods, may not be sufficient to limit recurrence during this vulnerable phase. This phase can be referred to as the “transition phase,” characterized by the recovering of the still weakened plantar soft tissues, after skin epithelialization. Understanding this transition phase is critical to designing effective offloading interventions that mitigate recurrence risk.

The primary aim of this review is to explore the current knowledge on foot physiology and biomechanics around the transition phase from ulcer healing to remission. Specifically, this review surveys the existing evidence on the types and anatomical regions of DFUs, the mechanical properties of plantar skin pre‐ and post‐DFU development, and the best international practice for offloading interventions across various DM risk categories. This information may be useful to develop state‐of‐the‐art offloading solutions for the transition phase in people with DM.

## 2 Materials and Methods

A narrative review of the literature on DFUs and current prevention strategies to limit ulcer recurrence was performed using PubMed, including studies published up to December 2024.

### 2.1 Search Strategy

The following keywords, and their combinations, were used to create multiple search strings in PubMed to look for all the relevant literature available online: “diabetic foot”; “transition phase”; “remission phase”; “ulcer”; “recurrence”; “recurrent”; “ulcer healing”; “footwear”; “offloading modalities”; “biomechanics”; “tissue properties”; and “plantar soft tissue.”

### 2.2 Inclusion and Exclusion Criteria

This review included studies on DFU recurrence, with a focus on the period immediately after ulcer healing (i.e., the “transition phase”), the physiology of ulcer healing in the later stages of wound closure and early remodeling, the efficacy of foot offloading modalities, and the biomechanical properties of plantar soft tissues in people with DM. Case reports, pilot studies, conference abstracts, editorials, commentaries, and thesis dissertations were excluded. Studies without clear or robust methodologies, such as insufficient reporting (e.g., skipping key details about the population, outcome or intervention), weak analysis (e.g., missing a rationale for poor sample size taken, missing a statistical analysis where feasible and others) were also excluded from the review process.

### 2.3 Eligibility Assessment

Two authors (K.S. and H.S.) with research backgrounds in DM independently screened the titles and abstracts for duplicates and for adherence to inclusion and exclusion criteria. Any dispute regarding a study's inclusion/exclusion was discussed further with a third author (L.B.) with a background in clinical medicine to reach a final decision. The shortlisted studies were then assessed for eligibility by five authors: two early‐stage researchers (K.S. and H.S.) in the field of DM, two experienced researchers (P.C. and G.R.) in the field of lower extremity complications and biomechanics and one senior physiatrist (L.B.) with more than 20 years of clinical‐research experience. All the studies that met the eligibility criteria were included for full assessment. If any of the above authors were also a co‐author of the study under consideration, they were replaced by other authors for assessment. To minimize interpretation and reporting bias, the manuscript was revised by two additional authors (A.L. and S.B.), both senior researchers, working in the fields of DFUs and human movement biomechanics.

### 2.4 Review Topics

The papers included in the review were further divided into four main topics: types of DFU recurrence; foot regions at risk of recurrence; mechanical properties of the plantar soft tissue; and current practice for offloading modalities.

## 3 Results and Discussion

**FIGURE 1:**
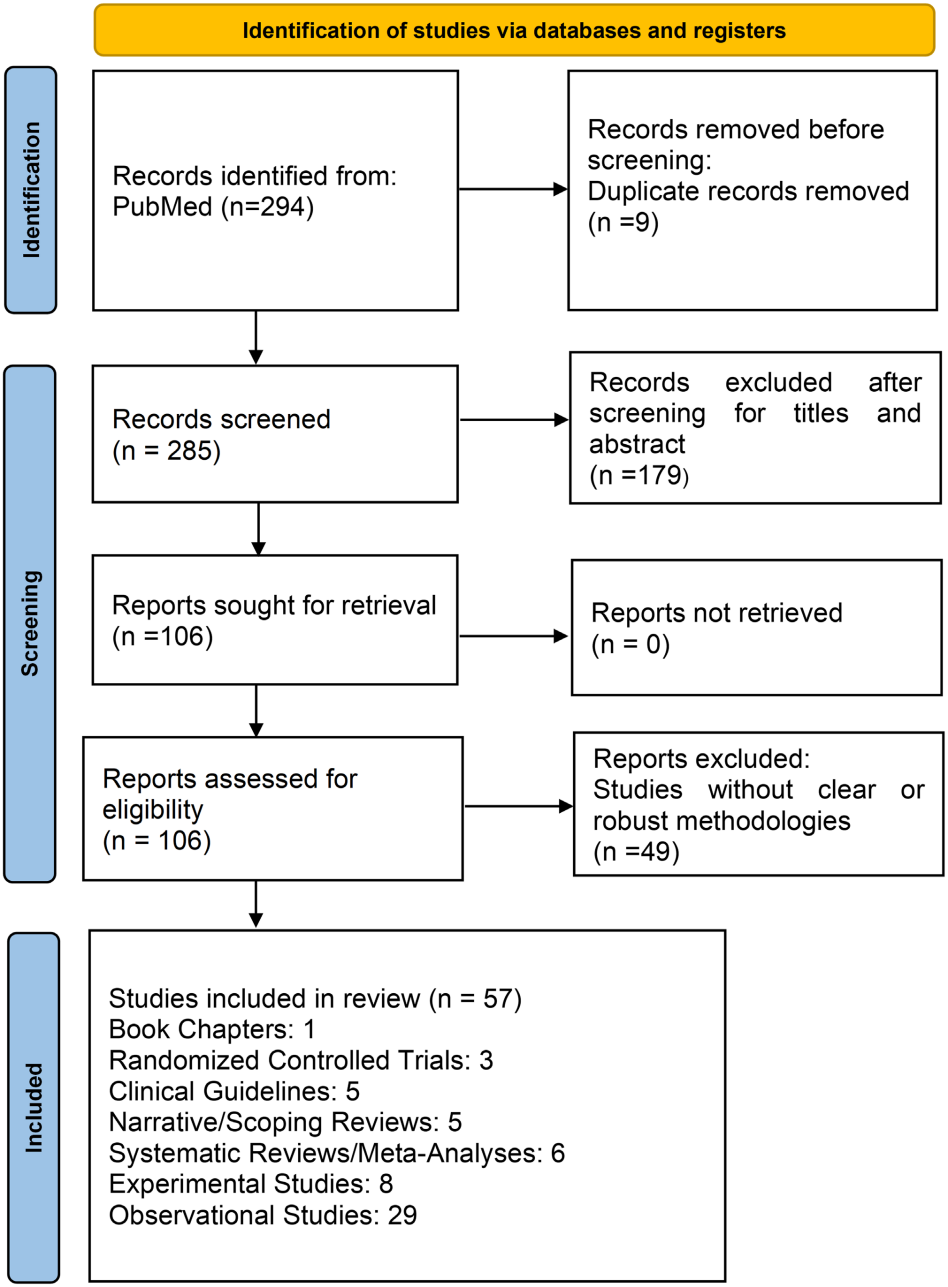
Flowchart showing the selection of studies in the review.

The literature review resulted in 294 papers matching the search criteria. Figure 1 illustrates the selection process by which 57 studies were included in the review. Study design and major outcomes of each included study are reported in Table 1.

**TABLE 1:** Studies included in the review and their major outcomes.

References	Main finding	Study design
[2]	DM causes histological changes in the skin.	Narrative/scoping review
[6]	Evidence‐based recommendations for offloading foot ulcers in people with DM.	Guidelines
[12]	DFU severity was linked to amputation, and microvascular issues to ulcer recurrence.	Observational (prospective, multicenter)
[13]	~40% of people experience recurrence within 1 year after ulcer healing, with almost 60% within 3 years and 65% within 5 years.	Narrative/scoping review
[14]	When worn as recommended, offloading‐improved custom‐made footwear significantly reduces DFU recurrence.	RCT (multicenter)
[15]	~48% of DFUs recurred on the contralateral foot, 17% of prior ulcers recurred at the same anatomical location.	Observational (multicenter)
[16]	42% of participants developed new ulcers within 2 years, need for clear definitions of recurrence.	Observational (prospective)
[17]	The plantar soft tissues of the elderly with DM were significantly stiffer and thinner compared to non‐diabetic.	Observational
[18]	Plantar soft tissues were significantly stiffer in the DPN group than those in the control group.	Observational (cross‐sectional)
[19]	The stiffness of plantar soft tissue was increased in diabetic groups compared with the control.	Observational
[20]	Updated definitions and criteria for diabetes‐related foot disease.	Guidelines
[21]	Evidence‐based recommendations for preventing foot ulcers in people with DM.	Guidelines
[22]	The global recurrence rate of DFUs was 22.1% per person‐year.	Systematic review & meta‐analysis
[23]	~57.5% recurrence rate of DFUs patients with previously healed ulcers over a 3‐year follow‐up.	Observational (prospective follow‐up)
[24]	Frequent use of therapeutic footwear could lead to lower ulceration rates.	Experimental (prospective)
[25]	The ulcerated group had significantly lower heel pad relative stiffness compared to the non‐ulcerated group.	Observational
[26]	Clinical recommendation to use a shoe‐based total contact device for the transition phase.	Narrative/scoping review
[26]	Non‐plantar DFUs are more common than plantar ulcers in people with severe disease.	Observational (prospective)
[27]	Risk factors for the recurrence of DFUs included male gender, smoking, diabetes duration, past DFUs duration, plantar ulcers, PAD, and DN.	Meta analysis
[28]	~33% of participants with a healed DFU developed a new or recurrent ulcer within a year.	Observational (retrospective)
[29]	The weighted mean re‐amputation rates at 1, 3, and 5 years were 20.14%, 29.63%, and 45.72%, respectively.	Systematic review and meta‐analysis
[30]	Recurrent ulcers were more likely to occur in the same foot and site as before, despite follow‐up and patient education.	Observational (prospective)
[31]	Plantar hallux ulcers were more likely to develop additional ulcers and recurrence at the same location.	Observational (prospective, cohort)
[32]	Recommendations for conducting rigorous and consistent studies, to improve the quality of evidence in wound management.	Guidelines
[33]	Glycation‐induced cross‐links cause two‐dimensional biomechanical alterations detectable by multiaxial testing.	Experimental
[34]	Skin hardening is a feature of peripheral neuropathy.	Observational
[35]	Energy dissipation ratio was higher for people with DM compared to non‐diabetics.	Observational (comparative)
[36]	Skin thickness and hardness were higher at ulcer sites compared to non‐ulcerated sites.	Observational
[37]	It's more likely that the injury in diabetic feet initiates in deeper tissue layers rather than on the skin surface, with tissues under the medial metatarsals being most vulnerable.	Experimental (computational model‐based)
[38]	Older adults had significantly stiffer forefoot plantar soft tissue at the 2 MTH compared to younger adults.	Observational (comparative)
[39]	The stiffness of plantar soft tissues increases with age, particularly at the big toe, MTHs and heel.	Observational
[40]	Increasing bodyweight loading from 0% to 80% causes the thickness of the heel plantar soft tissue to decrease by 12.0% and stiffness to increase by 83.4%	Observational
[41]	The plantar soft tissue homogeneity was greater in ulcerated than in non‐ulcerated people with DM.	Observational
[42]	MTH angle (hammer toe deformity) was the predictor of peak plantar pressures in individuals with DPN.	Observational (cross‐sectional)
[43]	Compared to irregular wear, regular wear of cushioned therapeutic shoes significantly reduced intercurrent foot lesions.	Observational (cohort)
[44]	Rigid rocker sole reduces the recurrence rate of plantar DFUs compared to a semi‐rigid rocker.	RCT
[45]	95° apex angle, 60% apex position, and 20° rocker angle is an optimal design for balancing offloading across different forefoot regions.	Experimental (randomized, controlled)
[46]	Paid employment, current or previous DFUs, satisfaction with follow‐up care, self‐efficacy, and specific storage practices are the factors associated with higher adherence to therapeutic footwear.	Observational (cross‐sectional)
[47]	Adherence to wearing custom‐made footwear was significantly lower for at home (61%) compared to away from home (87%), while overall rate was 71%	Observational
[48]	Custom‐made offloading modalities are more effective than standard devices for preventing DFUs.	Systematic review
[49]	The role of footwear in the prevention of complications related to diabetic foot.	Book chapter
[50]	Rigid outsoles lead to greater stride length and step velocity as compared to semirigid.	Experimental (cross‐sectional)
[51]	With adequate offloading, noninfected, nonischemic neuropathic plantar forefoot ulcers can heal in 6 to 8 weeks.	Narrative/scoping review
[52]	Total Contact casts (TCCs) have a shorter healing time compared to Removable Cast Walkers (RCWs) and half‐shoes.	RCT
[53]	Foot education, therapeutic footwear, and dermal thermometry are preventive measures for reducing the risk of DFUs.	Systematic review
[54]	Advanced age, male, elevated BMI, prolonged duration of diabetes, comorbid nephropathy, comorbid neuropathy, comorbid retinopathy, elevated systolic blood pressure, elevated FBS, elevated HbA1c, elevated triglycerides, elevated fibrinogen, elevated WBC count, elevated CRP, decreased ankle‐brachial ratio, decreased total protein levels are DFU risk factors.	Systematic review & meta‐analysis
[55]	The average annual incidence rate of new DFUs was 2.2%, in North‐west England.	Observational (prospective)
[56]	Depression is a risk factor for developing first foot ulcers in people with DPN, but not for recurrence.	Observational (prospective)
[57]	VPT was the strongest predictor for DFUs, with a relative risk of 25.4.	Observational (prospective)
[58]	Chronic kidney disease and DFU development and lower‐extremity amputations are associated with people with DM.	Observational (cohort)
[59]	Elevated pulse pressure is an independent predictor of DFUs.	Observational (cohort)
[60]	A close fit, lightweight, substantial tread, and a firm sole are design recommendations for enhanced balance.	Observational (Interview)
[61]	Guideline emphasizes the importance of footwear that fits, protects, and accommodates the shape of the feet to prevent foot ulceration.	Guideline
[62]	Rigid rockers are more effective for reducing pressure at the forefoot (MTH2‐5), while flexible rockers might be better for the first toe.	Experimental (randomized, controlled)
[63]	Insoles with medial arch and heel cup reduce PPP and PTI at medial arch and heel regions.	Experimental (crossover)
[64]	A full‐length shoe with a total‐contact inserts and an RRB sole is effective in reducing pressures on both the distal residuum and the contralateral extremity.	Experimental (prospective)
[65]	DFUs affect up to 68 per 1000 people with DM per year in the US, with DN, deformity, and repetitive stress, with non‐healing wounds are associated with wound depth, infection, and ischemia being the prominent risk factors.	Narrative/scoping review

Abbreviations: BMI‐Body Mass Index; CRP‐c‐reactive protein; DN‐Diabetic Neuropathy; DPN‐Diabetic Peripheral Neuropathy; FBS‐Fasting Blood Sugar; WBC‐White Blood Cell; PAD‐Peripheral Artery Disease; PPP‐Peak Plantar Pressure; PTI‐Pressure Time Integral; SWF‐Semmes‐Weinstein monofilament.

The number of papers relevant to the four main topics was the following:

- Types of DFU recurrence (*n* = 16);
- Foot regions at risk of recurrence (*n* = 13);
- Mechanical properties of the plantar soft tissue (*n* = 13);
- Current practice for offloading modalities (*n* = 23).

Despite the continuous effort by the IWGDF to share and promote guidelines for DFU prevention, the recurrence rate of DFUs remains high. A recent systematic review and meta‐analysis revealed that the global recurrence rates of DFUs over the past two decades have been constant at around 22% per person year, with only minor improvement since 2008 [22]. In a multicenter European study involving the Eurodiale study group, an approximate 20% recurrence rate was reported within the first few months of ulcer healing, which reached up to 60% over 3 years despite regular follow‐ups and patient education [23]. Similarly, another study on the clinical efficacy of therapeutic footwear in persons with DM at moderate to high risk of DFUs reported that most participants experienced DFU recurrence within the first 4 weeks of recruitment [24]. A similar trend was reported by Armstrong and colleagues [13] from the analysis of 19 studies, showing that the rate of change of DFU recurrence is the highest in the initial months after healing. This may be due to biomechanical factors, such as elevated pressure due to inadequate offloading strategies at this stage. In fact, persistent loading on less resilient plantar soft tissue areas can lead to microtrauma [25], which may go unnoticed due to diabetic neuropathy, eventually resulting in DFU recurrence. It appears, therefore, important to investigate and optimize offloading solutions for this phase right after DFU healing, as this could play a crucial role in reducing the rate of DFU recurrence. This ‘transition phase’ of DFU can be defined as a critical period that begins immediately after the DFU has been clinically considered healed (wound closure), when the foot is in remission. This phase, which is little investigated, represents a transition in the standard of care, where patients move from offloading strategies, used for DFU healing, to permanent long‐term footwear for ulcer prevention. The physiological duration of this phase is unknown, and clinicians often use their clinical judgment to limit it to around 4 weeks [66] after wound closure.

### 3.1 Types of DFU Recurrence

The number of people with DM in remission from a DFU is by far greater than those who have an active lesion [26]. The IWGDF defines a *recurrent foot ulcer* as a new DFU at any location on the foot, regardless of the location of the previous DFU [20]. However, authors have used different terminologies for recurring foot ulcers based on their location with respect to the position of the previous DFU. Terms such as *re‐ulceration* [67] or *other new foot ulcers* [16] have been used to refer to the generation of a new DFU at a different anatomical site than the previously healed one. This form of recurrence can be associated with the same underlying physiological and socio‐economic factors that contributed to the development of the initial DFU, such as peripheral neuropathy, gait‐altering foot deformity, lifestyle factors, and daily routines [27]. On the other hand, some authors have defined *recurrent ulceration* as the recurrence of the DFU at the exact location of the previously healed lesion [16, 28]. The underlying causes for this recurrence are usually mechanical factors such as high‐pressure loading of the still‐vulnerable tissue in the previously ulcerated area, residual gait and biomechanical alterations, or merely an incomplete healing of the previous DFU [15]. There is a lack of international consensus on using the above definitions, and authors often use them interchangeably [16]. The continued existence of varying terms in individual studies suggests an ongoing need for more granular definitions, particularly when investigating the precise mechanisms of recurrence and evaluating the efficacy of highly targeted interventions.

The majority of the reported DFU recurrences occur at new locations rather than at the original site, thus revealing their multifactorial pathogenesis [15, 16]. Moreover, studies only consider the first or the biggest lesion as the first episode and do not report on the other lesions that may occur before the first lesion is healed completely [15]. For these reasons, understanding the full scope of DFU recurrence and its etiology remains challenging. A clearer distinction between *re‐ulceration* and *recurrent ulceration* may improve diagnostic accuracy and tailor interventions more effectively. Understanding which type of recurrence is more prevalent or preventable during the transition phase will be crucial for developing targeted offloading and preventative strategies.

### 3.2 Foot Regions at Risk of Recurrence

**FIGURE 2:**
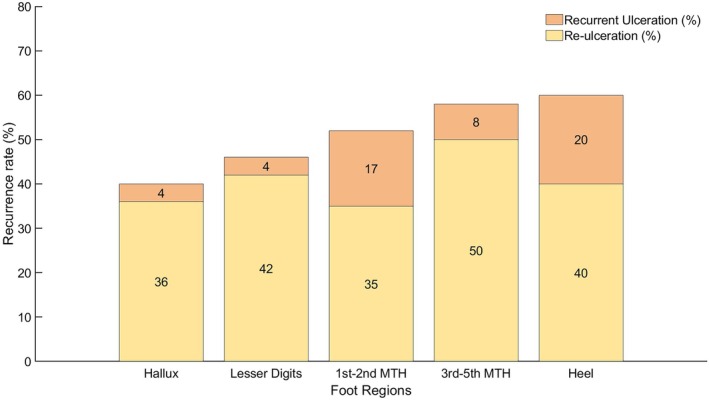
Percentages of re‐ulceration and recurrent ulceration rate out of a cohort of 129 participants with DM as reported in fig. 3 of Petersen et al. [15]. Each bar reports the percentage of the total wounds reported for that anatomical foot region. MTH stands for Metatarsal Head.

Several studies have reported that the recurrence rate is significantly higher in the first year of DFU healing compared to the second and third years [13, 23, 29]. A two‐year follow‐up study has shown that recurring DFUs are more prone to appear on the same foot than on the contralateral foot [30]. A similar trend was observed in a shorter 34‐week trial study [15]. While the importance of distinguishing the type of DFU recurrence has been highlighted [16], the location of new DFUs in relation to the previous or original ones is not always specified in the literature. A few studies [15, 16, 31] have reported on the recurrence rate of DFUs, categorizing them by anatomical location, and only one study [15] has reported along with the type of recurrence (see Figure 2). It can be observed that the rate of re‐ulceration (on a location different from the initial one) is higher than that of recurrent ulceration (on the same location) [15, 16]. It could be hypothesized that people with DFUs are often prescribed footwear to offload high‐risk areas, which, however, alters the normal gait pattern and results in more pressure on the contralateral foot. Although the risk of developing a plantar heel ulcer is low, literature shows that once a plantar heel ulcer has occurred and healed, the risk of developing another plantar ulcer, whether on the heel again or a different area, is higher compared to when the initially healed ulcer is at another location (Figure 2) [15]. For the hallux region, one study reports a high risk of re‐ulceration [15], while another reports a high risk of recurrent ulceration [31]. Overall, there appears to be a need for consistent terminologies and definitions to allow comparison of the results between different studies and to identify the true risk of DFU recurrence based on anatomical locations.

Therefore, research into the transition phase must prioritize the understanding of the biomechanical shifts and pressure redistribution patterns that contribute to the high rates of re‐ulceration, especially in the forefoot, during this critical post‐healing phase. This will enable the development of targeted offloading strategies and footwear interventions to mitigate early recurrence at specific anatomical locations.

### 3.3 Mechanical Properties of Plantar Soft Tissue

The IWGDF considers DFU as healed when the skin lesion is closed and does not present any drainage [20]. The European Wound Management Association Patient Outcome Group recommends confirming a healed wound after two consecutive visits, 2 weeks apart [32]. It has been reported that in people with DM, the epidermal layer of the plantar skin becomes thinner, and the plantar soft tissues become stiffer, particularly in the presence of neuropathy or previous ulcerations [19]. The collagen structure and function are altered in people with DM compared to healthy individuals [2, 33]. Compared with age‐matched healthy groups, the plantar soft tissue in people with DM neuropathy tends to be thicker [19], stiffer [19] and harder [34], with a trend for less energy return efficiency [35]. This reduces the tissue's cushioning properties, especially at the MTHs and heel pads, leaving them vulnerable to ulceration. This is consistent with the high rates of recurrent ulceration at the MTHs and heel region, as shown in Figure 2.

It has been proposed that wound formation may begin in deeper layers of soft tissues and not just on the skin surface [36, 37]. While a DFU is considered healed when the plantar skin is intact, as per the definition by IWGDF [20], the healing and recovery of skin and subcutaneous soft tissue continue over time, and therefore should be considered out of remission and fully recovered only when the plantar soft tissues regain their original mechanical properties or those of healthy plantar soft tissues in the same region.

**FIGURE 3:**
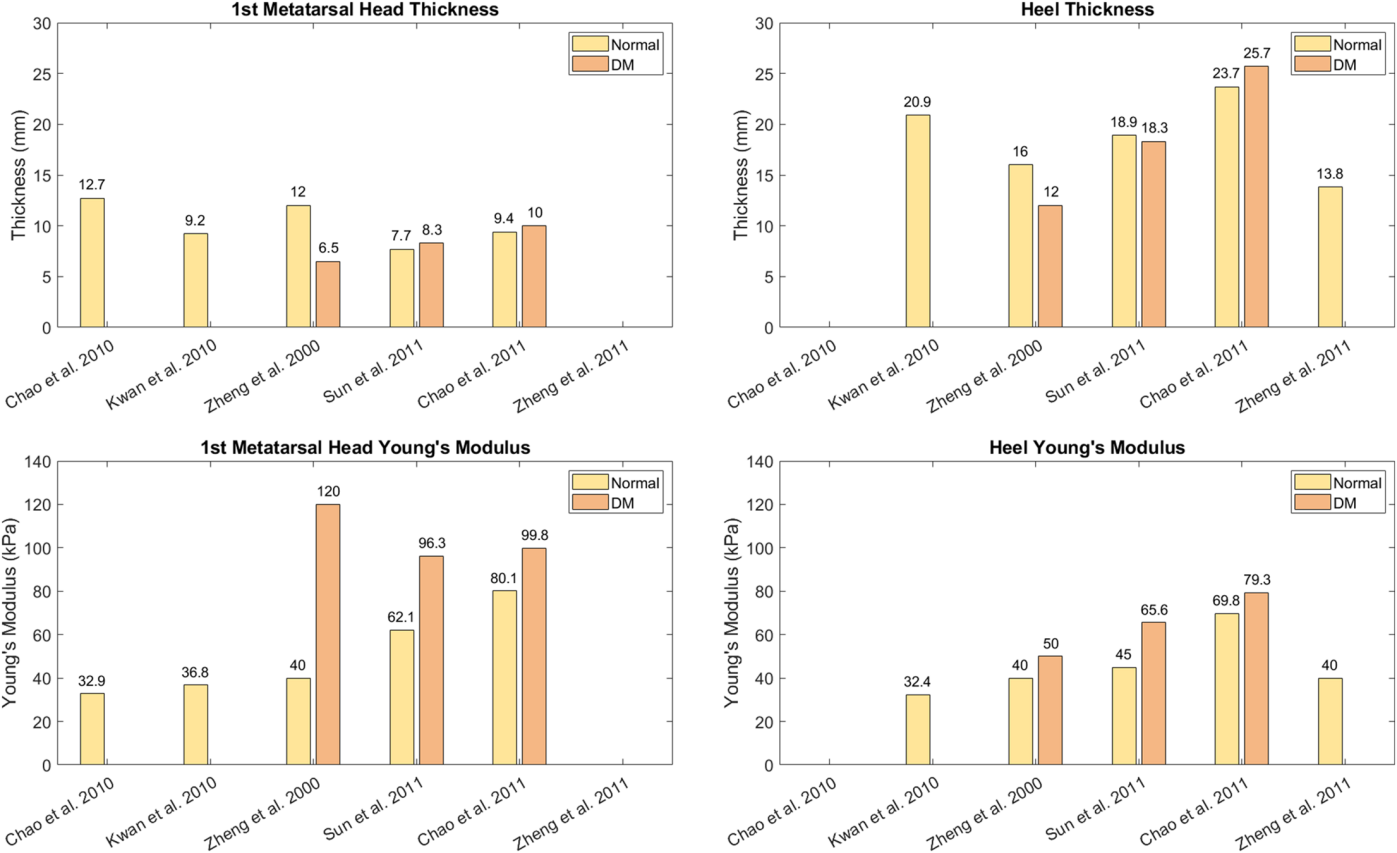
Young's modulus (kPa) and thickness (mm) of the plantar soft tissue at the 1st MTH and heel in people with and without DM and neuropathy reported in the literature [17, 18, 19, 38, 39, 40]. Data reported by Sun et al. is for people without DM and with DPN. Columns without a bar indicate that no data were reported for that category in the study. The reported data are from six cross‐sectional in vivo studies.

The two key mechanical properties that could help identify the remission status of a DFU are the local stiffness (in N/mm) or the Young's modulus (in kPa) and the thickness (mm) of the plantar soft tissue. Several studies aimed at estimating the in vivo mechanical properties of the plantar soft tissue of people with DM and neuropathy. By aggregating data from five in vivo studies [17, 18, 19, 38, 39], the Young's modulus of the plantar soft tissue at the first MTH of people without DM ranges 33–80 kPa (median 40 kPa). In people with DM, it ranges 96–120 kPa (median 99.8 kPa). Similarly, for the plantar soft tissue at the heel region, the stiffness coefficient ranges 32–70 kPa (median = 40.0 kPa) in people without DM, and 50–79 kPa (median = 65.6 kPa) in people with DM [17, 18, 19, 39, 40] (Figure 3). Unlike the plantar soft tissue, where the presence of DM is associated with an increase in the thickness, the epidermal layer of the plantar skin becomes thinner due to DM and neuropathy [19]; however, the results reported in the literature were not statistically significant. The variations in plantar soft tissue measurements reported in the literature, as shown in Figure 3, may be due to the different experimental protocols and instruments used across studies, as well as to variations in the size and demographics of the recruited participants.

While the mechanical properties of plantar soft tissues in people with DM have been extensively reported, the effect of DFUs and their healing on tissue properties has thus far been scarcely investigated. Using real‐time strain elastography, it has been shown that ulcerated heel pads have significantly lower stiffness than those in the contralateral foot [25]. However, this study could measure only the initial part of the tissue's stress–strain behavior due to the low loads applied; thus, the complete nature of the soft tissue mechanics may not have been captured with this technique. The average epidermal plantar skin is significantly thinner in people with DM and a history of ulceration by 15% as compared to age‐matched non‐diabetic people [19]. In another study, the plantar soft tissue of ulcerated forefeet has been shown to have higher uniformity and homogeneity than that of non‐ulcerated forefeet [41]. The thickness of the soft tissues has been identified as a precursor to elevated plantar foot pressure [42], and for people with a history of DFU, this thickness is increased as compared to people without DM [19], which in turn may lead to DFUs. Hence, this physiological alteration makes it difficult to prevent DFU recurrence. There is no clear and distinct evidence regarding the mechanical properties of soft tissues and epidermal skin in individuals with DM following DFU healing. Therefore, further research in this area is needed.

While epithelialization of the DFU may meet the criteria for clinical wound closure, complete tissue regeneration and restoration of native tissue properties have not yet been achieved. Moreover, the mechanical properties of the plantar soft tissue could be altered beyond the epithelialization process [19, 25], thus leading to an increased risk of recurrence. Consequently, although the DFU may appear healed and the wound is no longer open, the underlying tissue may still be vulnerable and in need of continuing care and support to prevent the DFU recurrence.

Therefore, characterizing the mechanical properties of plantar soft tissue and epidermal skin during the transition phase could be crucial for assessing full tissue recovery and guiding appropriate offloading strategies objectively. This deeper understanding will allow for the development of interventions that specifically address the altered tissue mechanics during this vulnerable phase, potentially leading to a reduction in DFU recurrence.

### 3.4 Current Practice for Offloading Modalities

Most research on footwear solutions for DFU in secondary prevention has focused on long‐term offloading strategies, rather than addressing the immediate post‐healing phase, when the risk of tissue damage is the highest. Frequent use of therapeutic [24] and cushioned therapeutic footwear [21, 43] was associated with lower ulceration rates, implying that continuous application over a prolonged time yields protective benefits. Footwear design features, such as rigid rocker soles, have been shown to reduce the rate of plantar DFU recurrence as compared to semi‐rigid shoes. However, the median follow‐up time was short, lasting only 26 weeks [44]. An optimal design with a 95° apex angle, 60% apex position, and 20° rocker angle was proposed to balance offloading across different forefoot regions [45]. Studies focusing on adherence to therapeutic footwear for people with DM show that factors such as a history of DFUs and patient satisfaction with follow‐up care can lead to higher adherence [46]. Overall, adherence to wearing custom‐made footwear is higher in away‐from‐home conditions compared to at‐home conditions [47]. Nevertheless, although the focus is on long‐term strategies, the existing literature could provide some valuable lessons for designing offloading modalities during the transition phase. First, when tailored to specific needs for offloading in high‐pressure areas of the foot, footwear can lower the recurrence rate. This is because custom‐made offloading modalities have been shown to lower the DFU recurrence compared to standard offloading solutions [21, 48]. Second, these offloading methods can quickly relieve affected tissues when worn consistently according to guidelines. Custom‐made shoes modified for pressure relief can noticeably reduce the DFU recurrence rate within just a few months of usage [14]. Clinicians also recommend the use of a shoe‐based total contact modality, such as a surgical shoe or an off‐the‐shelf offloading shoe with a total contact molded insole for 3–4 weeks just after the DFU is healed [66].

**FIGURE 4:**
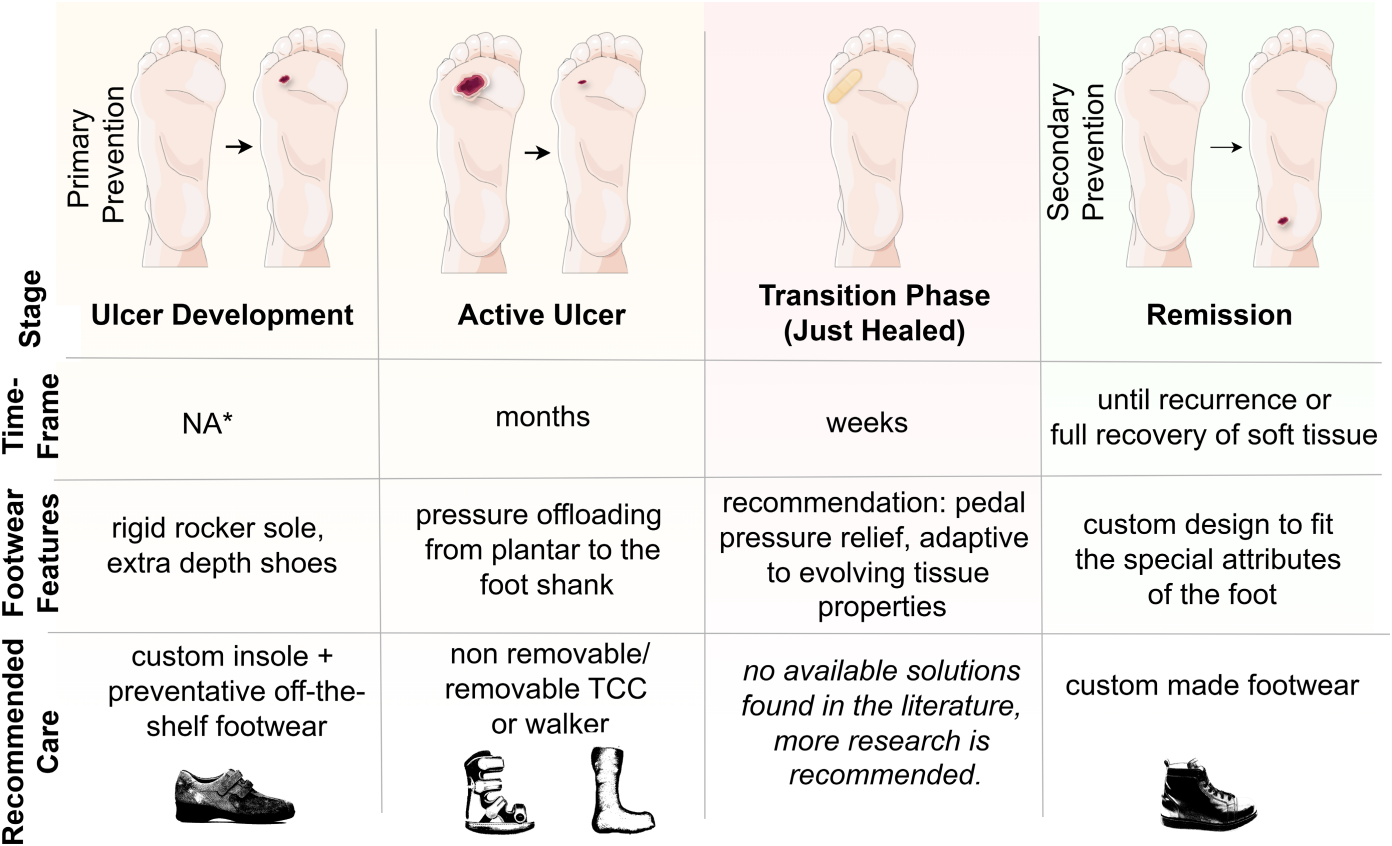
Multistage footwear strategies for DFU management [49]. *NA: not applicable. People with DM may only become aware of an issue once an ulcer is already clinically evident, meaning the developmental phase has an unobserved duration.

Therefore, a data‐driven, well‐designed offloading modality (possibly a custom‐made/semi‐custom‐made solution) focused solely on the transition phase can potentially bridge the gap (see Figure 4) between active wound care and sustained long‐term prevention management. Design clues for developing such offloading modalities could be based on footwear features recommended for different risk categories, as outlined in the IWGDF guidelines [21] and risk factors, both of which are presented in the later sections of this review.

#### 3.4.1 Risk Categories

Different footwear is recommended for individuals with DM based on the risk categories associated with developing DFUs [21]. The IWGDF categorizes diabetic feet into four risk categories. Risk categories 0–2 (very low risk–moderate risk) is for those people who have never had an ulcer and are recommended offloading modalities such as properly fitting footwear, extra‐depth shoes, custom‐made insoles, and toe orthoses, based on their increasing risk of mechanical stress due to factors like Loss of Protective Sensation (LOPS), Peripheral Artery Disease (PAD), and foot deformities. After ulceration or amputation along with LOPS or PAD, people with DM are classified in risk category 3 (high risk) of recurrence. Rigid rocker shoes, or those custom‐made to accommodate foot deformity or amputation, along with multilayered insoles, are effective in reducing relapse during remission [21, 49, 50].

For active ulcers, non‐removable knee‐high devices provide the most effective offloading for healing active plantar DFUs around the world [6, 51]. However, if not feasible, removable knee‐high or ankle‐high offloading solutions can also be used. It has been shown that total contact cast outperforms removable cast walkers and half‐shoes in healing DFUs and thus is still the gold standard for offloading during active DFUs when worn regularly [52].

Based on the progression of diabetic foot syndrome, the transition phase lies between the active ulcer stage and long‐term remission. Consequently, the offloading modalities in this phase should balance the intensive offloading used for active ulcers with the comfort provided by long‐term remission footwear.

#### 3.4.2 Risk Factors of DFU Recurrence

Research has been undertaken to identify various risk factors associated with the DFUs worldwide. Current risk stratification systems for DFUs typically incorporate variables consistently demonstrated in literature to be highly predictive of ulceration outcomes [53], such as LOPS, PAD, history of ulceration, to name a few. Notably, some studies have also shown a correlation between specific risk factors and potential indicators for the recurrence of DFUs. Demographic predictors like increased body mass index [53], and prolonged duration of diabetes [54] have been linked to the development of DFU. Predictors like higher age [55], biological gender male [53], and clinical depression [56] have been linked to both the DFU development and its recurrence; however, there exist no footwear solutions to reduce the risk of recurrence from these factors. Lifestyle‐based and metabolic‐related indicators like alcohol consumption [57], hypertension history [58], and systolic blood pressure [59] have been linked to the development of DFU but not to its recurrence [53]. Table 2 lists some of the most common independent risk factors linked to DFU recurrence, which could be mitigated by incorporating evidence‐based features in the footwear. Peripheral neuropathy in people with DM increases the risk of falling and ulceration due to a lack of protective sensation [44, 60]. Therefore, well‐fitting shoes with a proper cushioning effect have been shown to reduce the risk of recurrence of DFU [43]. Previous ulceration is a strong risk factor for recurrence [27, 31, 53, 54] and footwear features that aid in offloading plantar pressures from the region of previous DFUs can help reduce the risk of recurrent ulceration (DFUs at the exact location as that of the previous one). Behavioral factors, such as patient adherence to footwear recommendations, have been extensively studied [24, 46, 47] and identified as risk factors for recurrence. Research indicates that footwear designed to meet the aesthetic preferences [24, 46] of patients results in increased wear time, reducing plantar pressures, thereby decreasing the risk of recurrence.

**TABLE 2:** Risk factors for DFU recurrence and evidence‐based offloading strategies.

Category	Independent variables as risk factors for DFU	Footwear recommendations to mitigate risk
Neurological and sensory factors^a^	Peripheral neuropathy	Footwear with a close fit with tight fastening, lightweight construction, substantial tread, and a firm, molded sole/insole [60] to improve balance Therapeutic footwear with a rigid rocker sole [44] and cushioned insoles [43] to reduce the plantar pressure
	Foot deformity.	Prescription therapeutic footwear with custom‐made insoles to prevent recurrence [61]
Vascular factors^a^	Mild infections	Non‐removable knee‐high offloading devices [6]
Mechanical and biomechanical factors	Previous DFU at hallux region (a risk factor for re‐ulceration)	Forefoot Offloading Shoe (FOS) with a negative heel (half shoes) could help to shift pressure away from the hallux area, though it has been noted to be less comfortable and thus less adherence [21]
	Previous DFU at medial forefoot (a risk factor for re‐ulceration)	Stiff rocker shoes with an apex angle of 100 degrees [45]
	Previous DFU at the lateral forefoot (a risk factor for re‐ulceration)	Rigid rocker shoes with an apex position 50% from the rear of the shoe [62]
	Previous DFU at heel (a risk factor for re‐ulceration)	Orthosis with heel cup and medial arch features to reduce peak plantar pressure in the heel region [63]
	Previous lower limb amputation (transmetatarsal)	Full‐length shoe with total contact inserts and a rigid rocker bottom [64]
Adherence and patient factors	Poor patient adherence	Aesthetically pleasing [24, 47] and well‐fitting footwear [46]

^a^ Footwear recommendations for these risk factors may differ based on accompanying risk factors, such as foot deformities, a history of ulceration, or end‐stage renal disease, as these risk factors are cumulative in nature [65].

### 3.5 Limitations

This review has some limitations. First, only published literature available on PubMed was included in the review due to the focused biomedical scope. This may have omitted studies which were published only in print or another database. Second, only articles in the English language were included, thereby limiting the scope of the review. A narrative style was chosen for this review over the systematic review approach, the latter being a higher level of evidence, to provide a focused exploration of the transition phase concept and its implications for the standard of care in the remission phase. This enabled the current review to process more diverse perspectives around the transition phase rather than a strictly quantitative analysis of homogeneous and comparable data. Subjective bias in selecting relevant studies and in interpreting and reporting the findings may be present in the current study. This bias is mitigated by having a diverse panel of co‐authors from different clinical and research backgrounds, each with several years of experience in this field. Lastly, there is no consensus on the use of terminologies regarding the development of DFUs after the healing of the first lesion, and authors often use different definitions or no definitions at all [16]. Therefore, some studies may have been missed due to the use of a different terminology that does not appear in the search string, which could have been relevant to the scope of this review.

### 3.6 Research Implications

There is a lack of dedicated offloading solutions tailored for the transition phase. Further research is needed to define the characteristics of this crucial phase, since epidemiological studies reveal that the global recurrence rates remain high, especially during the initial months after healing. Moreover, the footwear requirements for this phase should be accurately identified to develop beyond state‐of‐the‐art footwear solutions. Future research should prioritize the design and validation of tailored footwear that incorporates pedal pressure relief, foot support mechanisms, and materials engineered to support the changing mechanical properties of the foot. Furthermore, to prescribe the best offloading solutions, the duration of this phase should be determined according to the physiological healing of soft tissues. Filling this gap in diabetic foot care may lead to a more structured and evidence‐based approach to managing the post‐healing phase, ultimately reducing the burden of DFU recurrence worldwide.

### 3.7 Clinical Implications

Clinicians are encouraged to examine how the mechanics of plantar soft tissues change due to DM and DFU. It is crucial to identify which evidence‐based footwear parameters have been shown to be effective, based on the risk category and the independent risk factors associated with developing DFU. These parameters, when combined with features such as adaptive pressure relief, real‐time pressure checks, and designs that focus on the patient's needs to protect the weak tissue, may lead to lower rates of recurrence during the transition phase.

## 4 Conclusion

According to the present review, epithelialization of the DFU site usually indicates clinical healing. However, DFUs can't be considered fully cured yet, as they enter a crucial transition phase that is often overlooked. There is no consensus in the literature regarding the terminology used to describe a recurrent foot ulcer based on the location of the previous plantar DFU. Additionally, recurring ulcers are more likely to appear at a location different from that of the previously healed one. The results from this review highlight the need for a fresh approach to developing state‐of‐the‐art offloading solutions that focus on the ‘transition phase’, accounting for pressure offloading requirements, evolving tissue properties and patient preferences and adherence.

## Author Contributions

K.S. contributed to the conceptualization of the study, data curation, formal analysis, methodology, validation, and visualization, and was involved in writing the original draft as well as reviewing and editing the manuscript. H.S. participated in conceptualization, methodology, and validation and contributed to the original draft and its review and editing. S.A.B. was responsible for funding acquisition, project administration, validation, and reviewing and editing the manuscript. G.R. helped with the methodology, provided supervision, validation, and review and editing. A.L. handled project administration, resources, supervision, validation, review, and editing. L.B. contributed to the project administration, supervision, review, and editing. P.C. was involved in conceptualization, formal analysis, methodology, supervision, validation, and the preparation, review, and editing of the original draft.

## Funding

This work was supported by the European Union's Horizon 2020 research and innovation programme under the Marie Skłodowska‐Curie grant agreement no. 101073533 (DIALECT: Diabetes Lower Extremity Complications Research and Training Network in Foot Ulcer and Amputation Prevention).

## Ethics Statement

The authors have nothing to report.

## Consent

The authors have nothing to report.

## Conflicts of Interest

The authors declare no conflicts of interest.

## Data Availability

All data generated or analysed during this study are included in this published article.
